# Characteristics of patients with chronic back pain who benefit from acupuncture

**DOI:** 10.1186/1471-2474-10-114

**Published:** 2009-09-21

**Authors:** Karen J Sherman, Daniel C Cherkin, Laura Ichikawa, Andrew L Avins, William E Barlow, Partap S Khalsa, Richard A Deyo

**Affiliations:** 1 Group Health Research Institute, Seattle, USA; 2 Division of Research, Northern California Kaiser Permanente, Oakland, USA; 3 Cancer Research and Biostatistics, Seattle, USA; 4 Division of Extramural Research and Training, National Center for Complementary and Alternative Medicine, National Institutes of Health, Bethesda, USA; 5 Deparment of Family Medicine, Oregon Health and Science University, Portland, USA

## Abstract

**Background:**

Although many clinicians believe there are clinically important subgroups of persons with "non-specific" low back pain, such subgroups have not yet been clearly identified. As part of a large trial evaluating acupuncture for chronic low back pain, we sought to identify subgroups of participants that were particularly responsive to acupuncture.

**Methods:**

We performed a secondary analysis of data for the 638 participants in our clinical trial comparing different types of acupuncture to usual care to identify baseline characteristics that predicted responses to individualized, standardized, or simulated acupuncture treatments. After identifying factors that predicted improvements in back-related function or symptoms, we determined if these factors were more likely to predict improvement for those receiving the acupuncture treatments than for those receiving usual care. This was accomplished by testing for an interaction between the prognostic factors and treatment group in four models: functional outcomes (measured by the Roland-Morris Disability Scale) at 8 and 52 weeks post-randomization and symptom outcomes (measured with a numerical rating scale) at 8 and 52 weeks.

**Results:**

Overall, the strongest predictors of improvement in back function and symptoms were higher baseline levels of these measures, receipt of an acupuncture treatment, and non-use of narcotic analgesics. Benefit from acupuncture compared to usual care was greater with worse pre-treatment levels of back dysfunction (interaction p < 0.004 for the functional outcome, Roland Morris Disability Scale at 8 weeks). No other consistent interactions were observed.

**Conclusion:**

This secondary analysis found little evidence for the existence of subgroups of patients with chronic back pain that would be especially likely to benefit from acupuncture. However, persons with chronic low back pain who had more severe baseline dysfunction had the most short-term benefit from acupuncture.

## Background

Low back pain is a common and costly problem that plagues the developed world [1-5]. Although there are a plethora of treatment options for low back pain, most evaluated treatments are of modest, if any, benefit [6,7]. One explanation for this observation is that there are, as yet, unidentified subgroups of persons with back pain who would be more likely to respond to some treatments than to others [8,9]. The challenge, then, would be to identify which individuals would be most likely to benefit from which treatments. In fact, most primary care clinicians believe there are distinct subgroups of patients within currently defined "non-specific" low back pain [9]. If true, determining which treatments would be most beneficial for specific types of patients could substantially improve the effectiveness and efficiency of treatment [10,11].

Although many studies have identified factors associated with improvement in back pain [12-14], only a few have looked at those which predict improvement from a specific treatment. For example, analyses by Underwood [15] suggested that greater expectations of treatment success might be more likely to lead to improved outcomes for patients receiving exercise combined with spinal manipulation than for those receiving usual care, exercise alone, or manipulation alone. Other studies found that persons with high levels of fear avoidance were more likely to benefit from an educational booklet and an exercise program [16] than were those without high levels of fear avoidance.

This study contributes to this nascent field of research by analyzing data from a large randomized trial of acupuncture for chronic low back pain [17] in order to determine if there are identifiable subsets of patients who were especially likely to benefit from acupuncture.

## Methods

We performed a secondary analysis of data for the 638 participants in our clinical trial of acupuncture for chronic back pain. In that trial, we found that participants who received acupuncture or simulated acupuncture had greater improvements in functional status and symptoms at the end of the treatment and at follow-up than those receiving usual medical care [17]. Details of the primary results and trial design are presented elsewhere [17,18], but a brief summary of the trial design is provided below. The trial was conducted in two integrated health care systems (Group Health in Seattle and Kaiser Permanente in Northern California) whose institutional review boards approved the study.

We recruited 638 participants 20 to 70 years of age with non-specific low back pain that had lasted at least 3 months. Persons with prior use of acupuncture were excluded. Participants were randomized to one of four treatments: individualized acupuncture, standardized acupuncture, simulated acupuncture (non-insertive stimulation of acupuncture points), or usual medical care. Participants were informed that the study was evaluating "different methods of stimulating acupuncture points". Those randomized to acupuncture or simulated acupuncture received 10 treatments over 7 weeks. All participants also received a self-care book and retained full access to the medical care provided by their insurance benefit. Telephone interviewers, masked to treatment, administered questionnaires to participants at baseline and at 8 and 52 weeks post-randomization.

### Baseline Data Collection

We collected baseline information on sociodemographic characteristics, status of current back pain, back pain history, health status, perceived likelihood of self-managing future back pain, and expectations of acupuncture's helpfulness. Back pain history included pain duration and prior use of injections, hospitalization or surgery for back pain. Participants who reported use of any of these three treatments were labeled as having received "intensive treatment". To characterize the current episode, we asked about activity limitations (i.e., the number of days spent in bed, lost from work or school, or cutting down on usual activities due to back problems during past month) [19], back-related functional status (using the 23-item modified Roland Morris Disability Scale [Roland score], where a higher score indicates greater dysfunction) [17,20], bothersomeness of current back symptoms using a 0 to 10 numerical rating scale (where a higher score is associated with worse symptoms) [17] pain below the knee, and medication use in the past week. The SF-36 Mental Health Component Summary Score was used to measure mental health status [21]. Finally, expectations of acupuncture's helpfulness was assessed using a 0 to 10 numerical rating scale, which were then analyzed in three categories: top tertile of expectations (8-10), lower two tertiles of expectations (0-7), or "could not rate" for those who could not provide a rating. The characteristics of the study population are presented in Table 1.

**Table 1:** Baseline characteristics of study population

	**Individualized acupuncture**	**Standardized acupuncture**	**Simulated acupuncture**	**Usual care**	**Total**
**Characteristic**	**(n = 157)**	**(n = 158)**	**(n = 162)**	**(n = 161)**	**(n = 638)**
**Demographics**					
Age, mean (s.d.)	47 (13)	49 (13)	47 (14)	46 (13)	47 (13)
Gender, % Female	68	56	60	64	62
Education, % College graduate	49	57	56	51	53
Employment					
% Not employed*	22	21	22	19	21
% Sedentary job	32	31	30	31	31
% Light or medium lifting job	31	33	31	33	32
% Heavy lifting job	15	15	18	17	16
					
**Back pain history and current episode**					
Roland score (0-23), mean (s.d.)	10.8 (5.2)	10.8 (5.6)	9.8 (5.2)	11.0 (5.2)	10.6 (5.3)
Bothersomeness score (0-10), mean (s.d.)	5.0 (2.5)	5.0 (2.3)	4.9 (2.4)	5.4 (2.4)	5.1 (2.4)
Duration of chronic low back pain, % 1+ year(s)	69	74	60	70	68
Number of low back pain days in past 3 months, mean (s.d.)	68 (26)	73 (22)	70 (24)	73 (23)	71 (24)
Disability days**, % any	42	43	40	43	42
Pain travels down knee, % Yes	21	22	22	21	21
Prior injection, surgery, or hospitalization for low back pain, % Yes	12	10	10	11	11
Any medication use in past week					
% None	38	38	37	35	37
% Narcotics	11	13	11	13	12
% Other	51	49	53	52	51
					
**Mental health**					
SF-36 Mental Health component score, mean (s.d.)	53 (8)	54 (8)	54 (7)	53 (8)	53 (8)
					
**Self-efficacy and Expectations**					
Likelihood of self-managing future back pain, % Very likely	3	2	8	6	5
Expectation of acupuncture helpfulness (0-10 scale)					
% Lower two tertiles (0-7)	57	53	56	49	54
% Top tertile (8-10)	25	24	28	33	28
% Could not rate	18	23	16	18	19

* Includes homemaker, student, unemployed, and retired.

** Any days spent in bed, lost from work or school, or cutting down on usual activities due to back problems during past month.

### Statistical Methods

Prior to undertaking the analysis, we identified 15 variables as potential predictors of change in outcomes. These were four demographic measures, three questions about back pain history, five questions about current back pain episode, one scale about mental health status, and one question each about likelihood for self-managing future back pain and expectations of acupuncture. Sociodemographic information included age, gender, educational level (trichotomized as at least college graduate vs. other vs. unknown) and physical demands at work (categorized as unemployed, sedentary, light/medium lifting, heavy lifting, unknown). Back pain history included length of time since first back pain (categorized as less than one year, a year or more, unknown), ever had any intensive back treatments (i.e., injection, hospitalization, or surgery), and number of days of back pain in the last 3 months. The questions characterizing the current episode of back pain included the baseline dysfunction score (Roland score), the baseline bothersomeness score, if there were any of 3 types of activity limitations in the last month described in the previous paragraph (yes vs. no), medication use in the past week (narcotics, any other, none,) and pain below the knee (yes vs. no). Mental health status was measured by the Mental Component Score of the SF-36 and self-efficacy and expectation of acupuncture helpfulness were categorized as reported in the previous paragraph.

Because important outcomes among persons with back pain include both functional improvement and symptom relief in the near and long-term, we constructed four separate ordinary least squares linear regression models to explore whether functional status or symptoms changed in response to acupuncture or simulated acupuncture treatment: dysfunction (Roland score) and bothersomeness score at both 8 and 52 weeks. These four initial models evaluated which of the 15 candidate variables predicted change in one of the outcomes at a specific time point. In addition to the 15 candidate variables described above, we included treatment group.

We then constructed reduced models for each of the four outcome/time points. These models included only those variables that were significant predictors of outcome at α ≤ 0.05 (for a two tailed test) in at least one of the initial models. We also chose, a priori, to include gender and expectation of acupuncture helpfulness because gender is often related to outcome and many other studies have found patient expectations for a treatment associated with better outcomes among those who received it [22-25]. The reduced models also included interaction terms between each treatment group and all other independent variables. This allowed us to test the hypothesis that specific predictor variables could predict response to different acupuncture treatments. Because of the large number of comparisons, we only report interactions where α ≤ 0.01 for a two tailed test. Furthermore, we looked for consistency of the interactions among both acupuncture groups and the simulated acupuncture group to rule out spurious results that might occur for one group, but not the other acupuncture groups.

Finally, we decided to test whether the percentage change in the dysfunction outcome at 8 weeks was constant over the range of baseline values for each of the three acupuncture or simulated acupuncture groups. We used a log transformation of our data, first adding a constant value of 1 to the dysfunction score to eliminate values equal to zero. Therefore, the model we tested was: log_10 _[(Roland at 8 weeks + 1)/(Roland at baseline + 1)] = (log_10_(Roland at 8-weeks + 1) - log_10_(Roland at baseline + 1)). All data were analyzed using SAS/STAT version 9.1 [26].

## Results

### Baseline Predictors of Outcome

Treatment group and six of the 15 potential predictor variables were significant predictors of changes in outcome in at least one of the four models (Tables 2 and 3). Treatment group and baseline dysfunction score were significant in three of the four models. Persons receiving acupuncture or simulated acupuncture improved more in function than those who received usual care at both 8 and 52 weeks and in symptom reduction at 8 weeks. Higher baseline dysfunction scores were associated with worse dysfunction scores at both 8 and 52 weeks and with worse bothersomeness scores at 52 weeks. Higher baseline bothersomeness scores predicted higher bothersomeness scores at both 8 and 52 weeks. Use of narcotics was associated with worse functional and symptom outcomes at 52 weeks. No other measures were found to be significant predictors of outcome in more than one of the four models.

**Table 2:** Multivariate analysis of overall predictors of back related dysfunction (Roland score)

		Roland score*			
		
		**8-week**		**52-week**	
**Variable**	**Categories**	β ** (SE)	**P-value**	β ** (SE)	**P-value**
Baseline Roland score	Continuous	**0.43 (0.05)**	**<.0001**	**0.56 (0.05)**	**<.0001**
Baseline bothersomeness score	Continuous	0.10 (0.10)	0.30	0.001 (0.10)	0.99
Any disability	Yes vs. No	0.78 (0.46)	0.09	0.01 (0.49)	0.99
SF-36 Mental Health score	Continuous	-0.02 (0.01)	0.17	-0.01 (0.01)	0.58
Age	Continuous	-0.01 (0.02)	0.51	0.02 (0.02)	0.33
Gender	Female vs. Male	0.42 (0.41)	0.32	0.37 (0.44)	0.39
Education level	College graduate, Yes vs. No	-0.19 (0.42)	0.65	-0.81 (0.44)	0.07
Employment	Heavy lifting job vs. Unemployed	-0.16 (0.70)	0.81	-1.76 (0.74)	0.16
	Light/Medium lifting job vs. Unemployed	-0.53 (0.57)		-1.22 (0.60)	
	Sedentary job vs. Unemployed	-0.61 (0.59)		-0.86 (0.63)	
	Employment unknown vs. Unemployed	-1.07 (1.84)		-0.93 (1.90)	
Medication use	Narcotics vs. None	0.43 (0.69)	0.81	1.77 (0.73)	0.04
	Other vs. None	0.02 (0.43)		0.12 (0.46)	
Self-efficacy	High vs. Low	0.61 (0.93)	0.51	-0.20 (1.00)	0.84
Expectation of acup. help	Top tertile vs. Lower two tertiles	-0.58 (0.47)	0.42	-0.23 (0.50)	0.55
	Could not rate vs. Lower two tertiles	-0.44 (0.53)		0.45 (0.56)	
Duration of chronic LBP	>= 1 y vs. <1 y	0.24 (0.45)	0.60	0.49 (0.48)	0.30
Pain travels below knee	Yes vs. No	-0.02 (0.51)	0.98	-0.37 (0.54)	0.50
Days of LBP in last 3 mo	Continuous	0.02 (0.01)	0.10	0.01 (0.01)	0.46
Intense LBP treatment	Yes vs. No	1.20 (0.63)	0.06	0.51 (0.66)	0.44
Treatment group	Individualized acupuncture vs. Usual care	**-2.38 (0.56)**	**<.0001**	**-2.21 (0.59)**	**0.001**
	Standardized acupuncture vs. Usual care	**-2.44 (0.55)**		**-1.81 (0.58)**	
	Simulated acupuncture vs. Usual care	**-2.86 (0.55)**		**-1.03 (0.58)**	

* For ease of identification, all independent variables with P < 0.5 are in boldface type

** The parameter estimates β refer to the amount of change in the outcome that is based on a one unit change in that covariate (continuous variables) or a change in category (categorical variables)

**Table 3:** Multivariate analysis of overall predictors of Symptom Bothersomeness score

		Symptom Bothersomeness*			
		
		**8-week**		**52-week**	
**Variable**	**Categories**	β ** (SE)	**P-value**	β** (SE)	**P-value**
Baseline Roland score	Continuous	0.02 (0.03)	0.37	**0.10 (0.03)**	**0.0004**
Baseline bothersomeness score	Continuous	**0.25 (0.05)**	**<.0001**	**0.17 (0.05)**	**0.001**
Any disability	Yes vs. No	0.27 (0.24)	0.26	-0.03 (0.25)	0.90
SF-36 Mental Health score	Continuous	-0.01 (0.01)	0.40	-0.01 (0.01)	0.26
Age	Continuous	-0.02 (0.01)	0.06	**0.02 (0.01)**	**0.03**
Gender	Female vs. Male	-0.14 (0.21)	0.50	0.05 (0.22)	0.81
Education level	College graduate, Yes vs. No	-0.04 (0.21)	0.87	-0.31 (0.22)	0.17
Employment	Heavy lifting job vs. Unemployed	**0.12 (0.35)**	**0.01**	-0.94 (0.37)	0.10
	Light/Medium lifting job vs. Unemployed	**-0.36 (0.29)**		-0.54 (0.31)	
	Sedentary job vs. Unemployed	**-0.86 (0.30)**		-0.67 (0.32)	
	Employment unknown vs. Unemployed	**-1.11 (0.94)**		-1.37 (0.97)	
Medication use	Narcotics vs. None	-0.10 (0.35)	0.22	**0.21 (0.37)**	**0.005**
	Other vs. None	-0.37 (0.22)		**-0.63 (0.23)**	
Self-efficacy	High vs. Low	0.07 (0.47)	0.87	**-1.04 (0.51)**	**0.04**
Expectation of acup. help	Top tertile vs. Lower two tertiles	-0.30 (0.24)	0.46	-0.22 (0.25)	0.55
	Could not rate vs. Lower two tertiles	-0.10 (0.27)		0.11 (0.29)	
Duration of chronic LBP	>= 1 y vs. <1 y	0.27 (0.23)	0.24	0.32 (0.24)	0.18
Pain travels below knee	Yes vs. No	0.43 (0.26)	0.09	-0.35 (0.28)	0.21
Days of LBP in last 3 mo	Continuous	0.004 (0.005)	0.35	0.001 (0.005)	0.91
Intense LBP treatment	Yes vs. No	-0.08 (0.32)	0.81	0.23 (0.34)	0.50
Treatment group	Individualized acupuncture vs. Usual care	**-1.05 (0.28)**	**<.0001**	-0.55 (0.30)	0.13
	Standardized acupuncture vs. Usual care	**-1.25 (0.28)**		-0.66 (0.30)	
	Simulated acupuncture vs. Usual care	**-1.39 (0.28)**		-0.47 (0.30)	

* For ease of identification, all independent variables with P < 0.5 are in boldface type

** The parameter estimates β refer to the amount of change in the outcome that is based on a one unit change in that covariate (continuous variables) or a change in category (categorical variables)

### Interaction between Baseline Predictor Variables and Treatment Response

**Figure 1:**
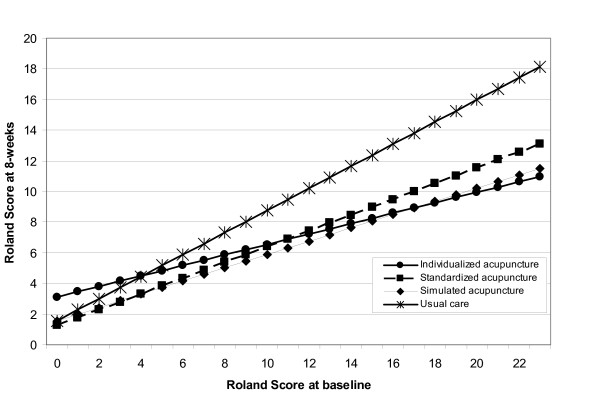
**Predicted values of the 8-week dysfunction score (Roland score) by baseline dysfunction score (Roland score) for each treatment group**. The predicted values are adjusted for baseline values of: Roland score, bothersomeness score, and age (as continuous variables); gender, employment type, medication use, acupuncture expectation, self-efficacy, and group (as categorical variables); and interaction between baseline Roland score and treatment group. The adjusted means assume a mean age of 47 years, bothersomeness = 5, and equal weighting in each level of the categorical covariates.

Compared to those receiving usual care, the 8 week dysfunction score improved more for those randomized to any of the three acupuncture or simulated acupuncture groups who had higher levels of dysfunction at baseline (overall interaction p = 0.004) (Table 4). There were consistent effects for each acupuncture group relative to usual care. A similar overall interaction between dysfunction and treatment was found for the 8-week bothersomeness score (interaction p = 0.01), but it was found in only two of the three acupuncture or simulated acupuncture groups, i.e., those receiving individualized or simulated acupuncture had greater improvement in the 8 week bothersomeness score if they had worse baseline back-related dysfunction (Table 5). There was no suggestion of an interaction in the standard acupuncture group, however. By 52 weeks, these interactions were no longer evident for either dysfunction or bothersomeness (Tables 4 and 5). The interaction between baseline dysfunction and acupuncture treatment appears to be due to an increased absolute improvement of the treatment groups compared to usual care when the baseline dysfunction score was worse. Figure 1 depicts the adjusted mean 8 week dysfunction score for each treatment group as a function of the baseline value of the dysfunction score. This figure clearly shows, as the baseline dysfunction score increases, the absolute difference between the 8 week dysfunction score of the acupuncture groups and the usual care group increases, with the acupuncture groups showing greater improvement in function. This is on an additive scale where the change in dysfunction score is the difference between the 8 week and baseline measures. However, when we measured the 8 week dysfunction score on a multiplicative scale in terms of the percent reduction from baseline, we found an approximately 30% reduction for the acupuncture groups across all levels of the baseline dysfunction score. This illustrates the importance of the scale used when determining whether there is statistical interaction between two variables. There were no other significant interactions between predictor variables and treatment group.

**Table 4:** Interaction between treatment group and significant baseline predictor variables for back related dysfunction (Roland score).

**Treatment**		Roland, 8-week*			Roland, 52-week*		
**group**	**Predictor variable**	β**	**SE**	**P-value**	β**	**SE**	**P-value**
Individualized acupuncture	Baseline Roland score	**-0.48**	**0.12**	**<.0001**	-0.23	0.13	0.07
	Baseline bothersomeness score	0.30	0.26	0.25	-0.13	0.28	0.64
	Age	-0.01	0.05	0.86	-0.02	0.05	0.64
	Gender	0.93	1.24	0.45	-0.68	1.30	0.60
	Employment, heavy lifting	4.29	1.97	0.03	**5.19**	**2.05**	**0.01**
	Employment, light/medium lifting	-0.34	1.62	0.83	2.18	1.69	0.20
	Employment, sedentary	1.34	1.67	0.42	2.73	1.76	0.12
	Medication use, narcotics	3.52	1.99	0.08	1.85	2.06	0.37
	Medication use, other	0.91	1.23	0.46	0.45	1.29	0.73
	Self-efficacy	-6.17	2.98	0.04	-3.46	3.46	0.32
	Acupuncture expectation, top tertile	-2.65	1.34	0.05	-0.98	1.42	0.49
	Acupuncture expectation, could not rate	1.43	1.57	0.36	-0.28	1.64	0.86
							
Standardized acupuncture	Baseline Roland score	**-0.37**	**0.13**	**0.004**	-0.24	0.13	0.07
	Baseline bothersomeness score	0.47	0.29	0.10	0.05	0.31	0.88
	Age	0.08	0.05	0.08	0.07	0.05	0.15
	Gender	0.90	1.19	0.45	1.57	1.25	0.21
	Employment, heavy lifting	3.00	1.97	0.13	3.03	2.08	0.15
	Employment, light/medium lifting	-0.84	1.62	0.60	-0.67	1.69	0.69
	Employment, sedentary	1.00	1.63	0.54	2.47	1.72	0.15
	Medication use, narcotics	2.20	1.91	0.25	4.06	1.98	0.04
	Medication use, other	0.46	1.24	0.71	1.21	1.30	0.35
	Self-efficacy	-2.91	3.32	0.38	1.67	3.49	0.63
	Acupuncture expectation, top tertile	-1.26	1.31	0.34	-1.90	1.38	0.17
	Acupuncture expectation, could not know	1.17	1.56	0.45	1.27	1.63	0.44
							
Simulated acupuncture	Baseline Roland score	**-0.41**	**0.13**	**0.001**	-0.07	0.13	0.62
	Baseline bothersomeness score	0.13	0.26	0.62	-0.19	0.27	0.48
	Age	0.02	0.05	0.65	0.05	0.05	0.33
	Gender	-0.48	1.22	0.69	-0.24	1.28	0.85
	Employment, heavy lifting	2.73	2.04	0.18	4.45	2.13	0.04
	Employment, light/medium lifting	0.02	1.66	0.99	-1.59	1.77	0.37
	Employment, sedentary	-0.85	1.72	0.62	1.57	1.82	0.39
	Medication use, narcotics	**4.81**	**1.96**	**0.01**	2.71	2.05	0.19
	Medication use, other	1.17	1.24	0.35	1.60	1.32	0.23
	Self-efficacy	0.19	2.25	0.93	1.97	2.42	0.42
	Acupuncture expectation, top tertile	-0.90	1.28	0.48	-2.91	1.35	0.03
	Acupuncture expectation, could not know	0.62	1.59	0.70	-0.17	1.66	0.92

* For ease of identification, all independent variables with P < 0.5 are in boldface type

** The parameter estimates β refer to the amount of change in the outcome that is based on a one unit change in that covariate (continuous variables) or a change in category (categorical variables)

**Table 5:** Interaction between treatment group and significant baseline predictor variables for Symptom Bothersomeness score

		**Bothersomeness,** 8-week*			**Bothersomeness,** 52-week*		
**Treatment group**	**Predictor variable**	β**	**SE**	**P-value**	β**	**SE**	**P-value**
Individualized acupuncture	Baseline Roland score	**-0.15**	**0.06**	**0.01**	-0.09	0.07	0.16
	Baseline bothersomeness score	0.04	0.13	0.74	0.02	0.14	0.87
	Age	0.03	0.02	0.23	0.01	0.03	0.60
	Gender	0.11	0.62	0.86	0.17	0.67	0.80
	Employment, heavy lifting	1.97	0.99	0.05	2.51	1.06	0.02
	Employment, light/medium lifting	-0.70	0.81	0.39	1.35	0.87	0.12
	Employment, sedentary	0.52	0.84	0.54	1.20	0.91	0.19
	Medication use, narcotics	0.35	1.00	0.72	0.14	1.06	0.90
	Medication use, other	0.36	0.61	0.55	-0.06	0.67	0.93
	Self-efficacy	-2.21	1.49	0.14	-1.52	1.78	0.40
	Acupuncture expectation, top tertile	-1.10	0.67	0.10	-1.44	0.73	0.051
	Acupuncture expectation, could not know	-0.27	0.79	0.73	-0.17	0.85	0.84
							
Standardized acupuncture	Baseline Roland score	-0.05	0.06	0.39	-0.06	0.07	0.39
	Baseline bothersomeness score	-0.06	0.15	0.69	-0.09	0.16	0.57
	Age	0.04	0.02	0.09	0.04	0.03	0.15
	Gender	0.38	0.60	0.52	0.78	0.64	0.23
	Employment, heavy lifting	0.87	0.99	0.38	0.68	1.07	0.53
	Employment, light/medium lifting	-1.28	0.81	0.12	-0.55	0.87	0.53
	Employment, sedentary	0.18	0.82	0.83	0.40	0.89	0.65
	Medication use, narcotics	1.01	0.96	0.29	0.44	1.02	0.67
	Medication use, other	0.07	0.62	0.91	0.05	0.67	0.94
	Self-efficacy	-1.82	1.66	0.27	-1.30	1.80	0.47
	Acupuncture expectation, top tertile	-0.68	0.66	0.30	-0.87	0.71	0.22
	Acupuncture expectation, could not know	0.35	0.79	0.66	0.70	0.84	0.41
							
Simulated acupuncture	Baseline Roland score	**-0.22**	**0.06**	**0.0005**	0.02	0.07	0.79
	Baseline bothersomeness score	-0.08	0.13	0.56	-0.10	0.14	0.46
	Age	0.04	0.02	0.07	0.05	0.03	0.08
	Gender	0.03	0.61	0.96	0.14	0.66	0.83
	Employment, heavy lifting	2.10	1.02	0.04	0.97	1.10	0.38
	Employment, light/medium lifting	0.70	0.83	0.40	0.05	0.91	0.96
	Employment, sedentary	0.07	0.86	0.94	1.03	0.94	0.27
	Medication use, narcotics	**2.49**	**0.98**	**0.01**	-0.19	1.06	0.86
	Medication use, other	0.70	0.62	0.26	-0.09	0.68	0.89
	Self-efficacy	-0.75	1.13	0.51	0.28	1.25	0.82
	Acupuncture expectation, top tertile	0.27	0.64	0.67	-1.29	0.69	0.06
	Acupuncture expectation, could not know	-0.16	0.80	0.84	0.75	0.86	0.38

* For ease of identification, all independent variables with P < 0.5 are in boldface type

** The parameter estimates β refer to the amount of change in the outcome that is based on a one unit change in that covariate (continuous variables) or a change in category (categorical variables)

## Discussion

This secondary data analysis found that persons with more severe pre-treatment back dysfunction demonstrated the greatest benefits from acupuncture or simulated acupuncture treatment, as measured by changes on the Roland score. Few other significant interactions emerged and none were consistent for both short and long term follow-ups. Regression to the mean is probably responsible for some of the greater improvement after 8 weeks in members of the usual care group with the worst back pain, given our finding that individuals in the usual care group improved more if their baseline dysfunction scores were worse. However, this phenomenon is unlikely to explain why the difference between usual care and acupuncture at 8 weeks increased as the baseline dysfunction scores increased. Thus, we have demonstrated interaction on an additive scale with the measurement of the Roland scores as absolute changes from baseline. This may merely reflect the greater opportunity for absolute change in those with higher baseline Roland scores. However, looked at from the perspective of relative percentage change from baseline, which is consistent with working on a multiplicative scale, the acupuncture (and simulated acupuncture) groups reduced their dysfunction approximately 30% more than did the usual care group - no matter what the baseline dysfunction score actually was. Thus, there was no interaction on the multiplicative scale. In fact, the measurement of interaction here, as is commonly found, depends on the scale that is being used.

Our findings are generally consistent with those of the few prior studies attempting to identify subgroups of individuals who respond best to specific treatments for back pain. Typically, these studies report few strong and consistent characteristics that identify subgroups of treatment responders for a specific intervention [15,27]. However, most of these studies are not large enough to identify all but the strongest interactions. In one of the largest pragmatic trials of acupuncture for chronic back pain including over 2000 patients, Witt et al [28] found that 3 of 9 evaluated characteristics of patients (younger age, worse baseline back dysfunction, more than 10 years of education) were effect modifiers indicating better response to acupuncture. One of the challenges in comparing results across studies is that studies typically assess a somewhat different list of possible characteristics as potential moderators of response to treatment.

Our finding that pre-treatment expectations did not predict response to specific types of acupuncture differs from the findings of previous researchers. Kalauokalani [22] and Linde [23] both found that more optimistic expectations of treatment led to better outcomes from acupuncture. Thomas's [29] results were more complex. She found no benefit of acupuncture over usual care for persons with positive beliefs about acupuncture, but found acupuncture more effective for those who were agnostic about its benefits.

The results of these trials cannot be directly compared to our study because of differences in the way the data were collected and analyzed. However, our finding that individuals who could not provide a rating of their expectation of acupuncture's effectiveness did not have worse outcomes clearly demonstrates that our findings are different than those of Linde [23]. In that study, participants who could not rate their expectation of acupuncture's effectiveness did worse than the others, who nearly always believed that acupuncture would be "effective" or "very effective". Given the variability in the findings across these studies, further research is needed to understand the different effects of pre-treatment expectations on outcomes of acupuncture care.

Our study has a number of limitations. For one thing, our study only explored characteristics of individuals that were predictive of superior outcomes for acupuncture (or a type of acupuncture) versus usual care. Conceivably, our findings may have differed had we used a different comparison group. We did not collect data on fear avoidance, which is associated with poor prognosis in some data sets [27]. If patients with higher levels of fear avoidance were less likely to improve from acupuncture, our lack of information on this variable would be a limitation of our study.

Our study was large and high follow-up rates. However, the samples sizes required to detect interactions must be four times larger than that required for detecting a main effect of similar magnitude [30]. Thus, we would be able to detect only large interactions.

Finally, as with all post-hoc analyses, the results must be interpreted with caution and need to be replicated in other data sets. We suspect that replication would best be undertaken in the context of a meta-analysis using individual patient level data from all included studies, as that would increase the sample size substantially [31]. The Acupuncture Trialist Collaboration, a new international collaboration among researchers to share data and conduct meta-analyses from large trials of acupuncture for pain, may be well-suited to conduct such analyses.

Researchers have employed two different approaches in their attempts to identify sub-groups of persons with low back pain that would benefit from specific treatments. Some studies, including ours, have searched for sub-groups using regression analyses to see what characteristics are associated with superior outcomes for specific treatments. Others have developed "clinical prediction rules" wherein patients are initially categorized into more homogenous groups based on clinical findings and pain history. Such rules can then be tested in studies where patients are given treatments that are matched to the type of treatment that is believed better able to address their underlying problem [32]. For example, Childs [11] used this approach to validate a clinical prediction rule for spinal manipulation.

Clinical prediction rules have yet to be identified for acupuncture. In principle, various Chinese medicine findings, including Chinese medicine diagnosis, might be useful for developing such a rule. In practice, however, progress in this area has been limited because there is typically poor diagnostic concordance among TCM practitioners [33,34] and because individual patients with chronic low back pain are often given multiple TCM diagnostic labels [34,35].

Thoughtful collaboration among practitioners and researchers may ultimately lead to the development of prediction rules that match patients to the most appropriate health care provider. Such collaborations are most likely to be fruitful if they initially focus on developing comprehensive models that incorporate the physiological underpinnings of the biopsychosocial model [36].

## Conclusion

This analysis found little evidence for the existence of subgroups of patients with chronic back pain that would be especially likely to benefit from acupuncture. The only statistically significant and consistent finding was that persons starting with greater back dysfunction improved the most from acupuncture or simulated acupuncture after 8 weeks, in terms of change score, although the percentage improvement from baseline was consistent all levels of baseline dysfunction. Future studies are needed to confirm the findings of this post-hoc analysis.

## Competing interests

The authors declare that they have no competing interests.

## Authors' contributions

KJS, ALA, RAD were involved in the conceptualization and design of the analysis involved in this report; LI and WEB conducted the analysis; all authors were involved in interpretation of the findings; KJS, DCC, LI and WEB drafted the manuscript and all authors read and approved the final manuscript.

## Pre-publication history

The pre-publication history for this paper can be accessed here:


